# Psychological adaptation mechanisms of athletes’ cognitive resilience in high-temperature environments

**DOI:** 10.3389/fpsyg.2026.1735923

**Published:** 2026-03-11

**Authors:** Huixiang Guan, Songchen Gao

**Affiliations:** 1 School of Physical Education, Jiangsu University of Science and Technology, Zhenjiang, China; 2 Department of Physical Education, Wuxi Taihu University, Wuxi, China

**Keywords:** challenge appraisal, cognitive resilience, high-temperature environment, latent profile analysis, psychological adaptation, team support

## Abstract

**Introduction:**

This study integrates the “Stress and Coping” theory with the “Ordinary Magic” model to propose a sequential “challenge appraisal -resource gain -cognitive resilience” framework. The framework aims to elucidate the psychological adaptation processes contributing to athletes’ cognitive resilience in high-temperature environments. The study specifically explores the mediating role of challenge appraisal in the relationship between psychological resources and cognitive resilience, as well as the moderating effect of team support on this relationship.

**Methods:**

Data were collected from 240 professional athletes via a questionnaire-based survey, capturing multidimensional psychological and contextual variables. The analysis utilized structural equation modeling (SEM), latent profile analysis (LPA), and moderated effect testing to assess the proposed mediation, heterogeneity, and moderation pathways.

**Results:**

Findings reveal that cognitive resilience in high-temperature environments is a dynamic process influenced by cognitive reappraisal and resource coupling. The study demonstrates that challenge appraisal mediates the relationship between psychological resources and cognitive resilience, with team support acting as a moderating factor.

**Discussion:**

These results provide empirical support for targeted psychological interventions and the development of team-support systems in sports involving thermal stress. Additionally, the findings offer a theoretical advancement in sports psychology by transitioning from a static trait-oriented approach to a more dynamic “individual-context” interaction paradigm. This shift highlights the complex nature of psychological adaptation mechanisms in extreme environments.

## Introduction

Against the dual backdrop of global warming and the increasing specialization of competitive sports systems, high temperatures have emerged as a persistent stressor within athletic ecosystems. For instance, during events such as the Tokyo Olympics and multiple World Athletics Championships, numerous athletes exhibited symptoms including cognitive slowing, attentional drift, and tactical errors under heat stress (Taylor et al., 2016; Racinais et al., 2015). Empirical evidence suggests that heat not only induces physiological strain—challenging thermoregulatory and metabolic functions—but also directly impairs cognitive processing and psychological adaptation, thereby constituting a critical determinant of both performance and safety.

Substantial research has documented physiological countermeasures—such as precooling techniques, hydration strategies, and workload management—that help preserve performance under thermal stress (Racinais et al., 2015; Périard et al., 2015). Yet, such approaches often neglect the complexity of psychological adaptation. Studies indicate that even under comparable core temperatures and hydration status, athletes display marked individual differences in attentional control, emotional regulation, and tactical execution (Schmit et al., 2017). These variations are largely attributable to psychological regulatory capacity, particularly the construct of cognitive resilience. Cognitive resilience refers to the ability to sustain goal-directed attention and cognitive stability—such as in decision-making and attentional control—under thermally stressful and competitive conditions. Its activation appears to hinge upon a critical cognitive-affective process: the reappraisal of heat stress as a manageable challenge rather than an insurmountable threat (Tomaka et al., 1997).

Nevertheless, prevailing research exhibits a tendency toward psychological reductionism, conceptualizing cognitive resilience as a static trait independent of environmental and physiological contexts. This perspective fails to capture its emergent and dynamic nature, which unfolds through real-time interaction with physiological states and team-level factors in ecologically valid settings. Experimental studies confirm that heat amplifies threat perception and negative cognitive bias, thereby accelerating the depletion of executive function resources (Shibasaki et al., 2017). In the absence of effective regulatory mechanisms, such depletion readily precipitates motivational disengagement and performance deterioration. Conversely, team-level social support—such as constructive coach feedback and coherent team norms—has been shown to mitigate the psychological burden of heat by fostering challenge appraisals and reducing the disruptive effects of thermal discomfort on executive control (Morgan et al., 2019). This implies that cognitive resilience operates not in isolation, but within a tripartite coupling system encompassing individual, situational, and organizational dimensions. Simultaneously, team-based social support has been empirically demonstrated to facilitate the reappraisal of thermal stress as a manageable challenge by offering both emotional buffering and instrumental assistance (Filho et al., 2014; Adie et al., 2012). These findings indicate that the development of cognitive resilience is contingent not only upon intra-individual psychological resources but also on the synergistic contributions of external team support, collectively constituting a dynamic adaptive framework best described as a “thermal effect–individual resources–team support” system.

Therefore, psychological adaptation to heat should be conceptualized as a dynamic and evolving process. Through continuous attentional regulation, emotional reappraisal, and executive control, athletes strive to stabilize performance under the combined influence of interoceptive signals, task demands, and team climate. Longitudinal investigations reveal that with repeated heat exposure, athletes transition from reactive compensatory efforts to proactive preventive strategies—demonstrating a psychological trajectory from vulnerability to resilience (Tattersall et al., 2012). This progression underscores the core function of cognitive resilience: to preserve the elasticity and functional continuity of the psychological system under resource constraints and heightened uncertainty.

To address these dynamics, Integrating “stress-coping” theory with the “ordinary magic” framework of resilience, we advance a “challenge appraisal–resource gain” dynamic model. This model transcends conventional physiological or psychological siloes, offering an integrative theoretical lens for understanding athletic performance in hot environments. This study investigates three core questions concerning athletes’ cognitive resilience in high-temperature environments: whether challenge appraisal mediates the link between psychological resources and cognitive resilience; how team support moderates the resource–resilience relationship; and whether distinct, psychologically differentiated subtypes of cognitive resilience exist within athletic populations. By addressing these questions, the research aims to systematically clarify the developmental pathways of cognitive resilience under thermal stress, thereby offering a scientific basis for advancing theoretical models in sports psychology and designing evidence-based interventions for competitive practice.

### Theoretical foundations and research hypotheses

**Figure 1:**
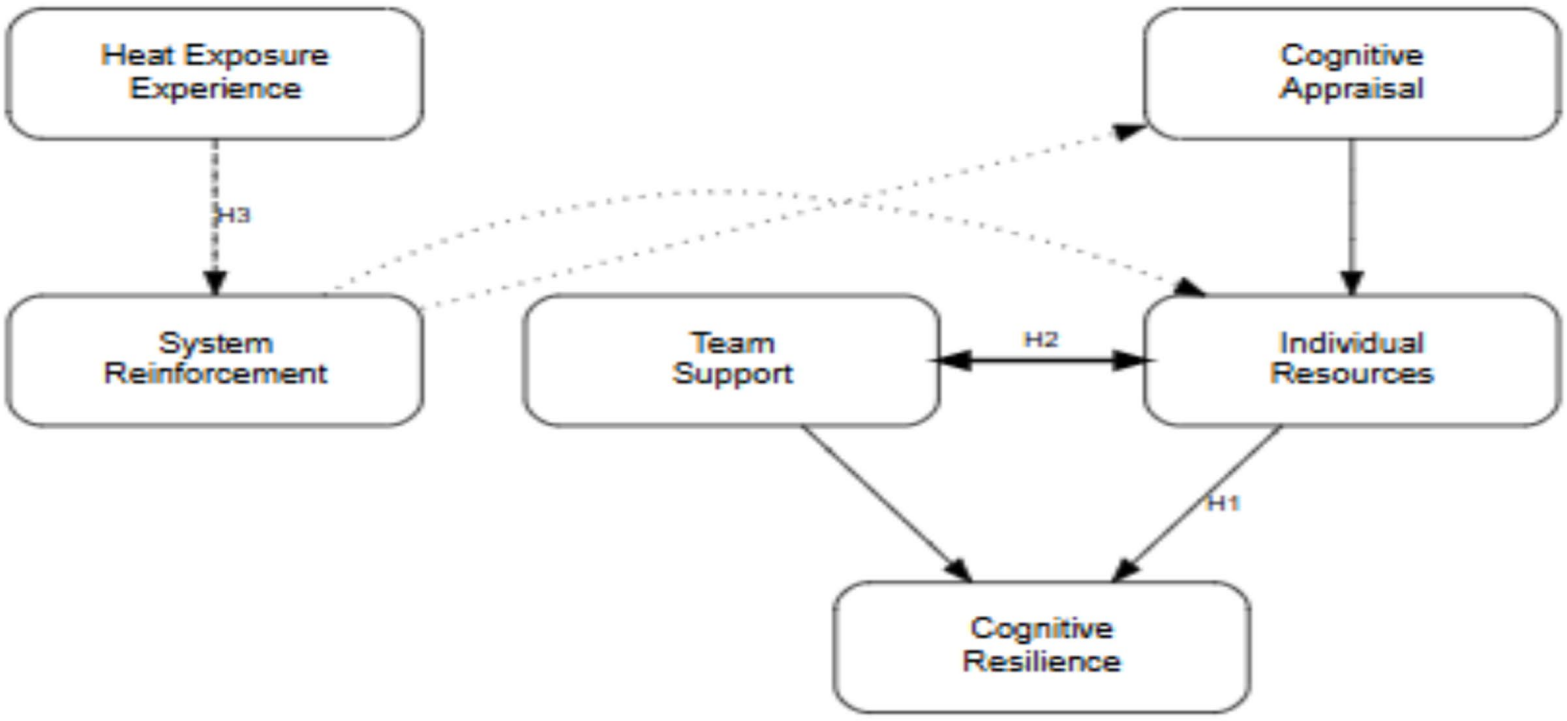
The challenge appraisal–resource gain model of cognitive toughness in high-temperature environments. The “Challenge Appraisal-Resource Gain” model integrates the Transactional Theory of Stress and Coping (defining the central role of challenge appraisal) with the Ordinary Magic Model (explaining the synergistic effects of individual resources and team support). Solid, dashed, and dotted arrows denote direct, indirect, and theorized long-term effects, respectively. H1–H3 indicate hypothesized paths.

To systematically elucidate the psychological adaptation mechanisms underlying athletes’ cognitive resilience in high-temperature environments, this study integrates Lazarus and Folkman's (1984) stress-coping theory with Masten's (2001) “ordinary magic” model to construct a dynamic “challenge appraisal-resource gain” framework (Figure 1). This integrated model transcends conventional approaches that examine physiological, psychological, or environmental factors in isolation, offering instead a comprehensive theoretical perspective.

As illustrated in Figure 1, the model delineates the formation mechanism of cognitive resilience under thermal stress. Cognitive appraisal initiates the coping process, wherein athletes reinterpret thermal stressors as challenges rather than threats. This appraisal triggers a dynamic interplay between individual psychological resources—including attentional flexibility and emotional regulation—and team-level support systems, which collectively sustain the emergence of cognitive resilience. Through repeated heat exposure, this adaptive system undergoes progressive reinforcement, evolving from reactive compensation to proactive prevention. Ultimately, this framework provides a systematic theoretical basis for understanding psychological adaptation in athletic high-temperature environments.

## Theoretical foundations

### Cognitive appraisal in high-pressure environments: from threat perception to challenge reconfiguration

The cognitive appraisal theory of stress and coping, introduced by Lazarus and Folkman (1984), conceptualizes stress as a process of subjective meaning-making, wherein cognitive resilience functions as the pivotal mechanism for transforming perceived threats into manageable challenges. According to this framework, individuals respond to stressors through a two-stage appraisal process: primary appraisal involves evaluating the situation’s relevance and potential threat to personal goals, while secondary appraisal entails assessing available coping resources.

In competitive sports, high temperatures act as a salient stressor that engages this two-stage cognitive mechanism. During primary appraisal, athletes interpret physiological cues—such as skin temperature exceeding 38 °C or heart rates surpassing 160 bpm—in relation to performance objectives like movement stability or tactical execution. This interpretive process is inherently subjective: some athletes sustain focused attention under identical thermal conditions by not categorizing heat as an uncontrollable threat (Racinais et al., 2015). In the secondary appraisal stage, athletes integrate multiple coping resources, including physiological self-efficacy developed through heat acclimatization training (e.g., interpreting profuse sweating as adaptive thermoregulation), structured strategies such as hydration protocols, and collaborative team behaviors like pacing adjustments. These resources collectively facilitate the reappraisal of thermal threats as achievable challenges, thereby reallocating cognitive resources from anxiety mitigation to task-oriented execution—for instance, refining technical skills such as cornering techniques. This process underscores the dynamic and constructed nature of cognitive resilience (Shibasaki et al., 2017).

Central to this adaptive process is challenge appraisal—the cognitive reframing of thermal stress as controllable rather than threatening. Successful cognitive restructuring serves as a critical prerequisite for the expression of high-level cognitive resilience. Performance variations among athletes under similar physiological strain can thus be attributed to differences in their capacity to reconstruct threatening stimuli as challenging opportunities—a phenomenon that cannot be fully explained by physiological adaptation alone. Enhanced resource perception during secondary appraisal, supported by emotional regulation and team-level cooperation, systematically sustains the real-time emergence of cognitive resilience.

In summary, the cognitive theory of stress and coping provides a “subjective meaning-making” framework for examining cognitive resilience in high-temperature athletic contexts. It refocuses scholarly attention from purely physiological metrics to the dynamic processes of psychological appraisal, clarifying that the core of cognitive resilience lies in “stress transformation capacity.” This theoretical lens addresses the question raised in the introduction regarding performance discrepancies under physiologically comparable conditions and establishes a conceptual foundation for subsequent investigation into the coupling of internal resources and external support.

### The dynamic emergence of cognitive resilience: coupling internal resources with external support systems

Masten's (2001) “Ordinary Magic” model elucidates the dynamic formation of cognitive resilience through the synergistic coupling of internal resources and external support systems during stress exposure. Departing from static trait-based conceptions of resilience, this framework emphasizes sustained individual-environment interactions as the fundamental driver of adaptive capacity, thereby shifting the focus from compensatory stress responses to proactive adaptation through integrated resource utilization and feedback regulation.

Internal support systems operate through attentional monitoring of physiological signals—such as cutaneous thermoreception and heart rate variability—enabling dynamic behavioral adjustments. For instance, distance runners may modulate stride frequency in response to rising core temperature to minimize metabolic load, or reappraise tachycardia as an adaptive cardiovascular response rather than a threat. Such regulatory processes liberate cognitive resources, redirecting attention toward tactical execution. External support systems, conversely, mitigate environmental uncertainty through structured interventions: coached heat-rotation strategies and standardized hydration protocols establish predictable behavioral frameworks, allowing athletes to allocate cognitive resources toward complex tasks such as game situation analysis rather than environmental management.

The integration of internal and external systems generates a self-reinforcing cycle of “capacity activation–resource empowerment,” enhanced through contextual cueing and leading to optimized cognitive resource allocation. This coupling manifests as an evolutionary trajectory from passive compensation to proactive prevention. Novice athletes often counteract thermal-induced attentional drift through effortful concentration, whereas experienced athletes, supported by internalized team strategies, develop preemptive cognitive routines. Beach volleyball players, for example, may incorporate thermally-attuned visual scanning rhythms into pre-match preparations, transforming environmental stress into kinesthetic guidance.

This progression demonstrates that cognitive resilience emerges through continuous person–environment interaction, enabling a functional shift from stress compensation to adaptive regulation. The core mechanism involves reallocating finite psychological resources from reactive thermal coping to proactive task optimization, thereby establishing a new psychophysiological equilibrium (Masten, 2018).

In summary, the “Ordinary Magic” framework provides critical theoretical grounding for understanding cognitive resilience under thermal stress. By delineating the dynamic coupling of internal regulation and external support, it models how cognitive resources are systematically reallocated from compensatory efforts to preventive optimization. This integrative perspective not only deconstructs the neuropsychological mechanisms of heat adaptation but also establishes an operational foundation for targeted psychological interventions through quantifiable modeling of individual–environment interactions.

### Resilience through systemic interaction: an individual-behavior-environment synergy

The development of cognitive resilience in athletes under high-temperature conditions emerges from dynamic, multi-level interactions among psychological traits, behavioral strategies, and environmental factors. Core psychological constructs such as self-efficacy (Bandura, 1977) and emotion regulation capacity (Gross, 2015) establish the foundational individual resources, while goal-directed training protocols (Locke and Latham, 1990) and stress-coping strategies (Lazarus and Folkman, 1984) function as critical mediators translating internal dispositions into performance outcomes. When athletes face prolonged heat exposure, environmental stressors may initially compromise cognitive function through hypothalamic–pituitary–adrenal (HPA) axis activation (Chrousos, 2009), yet systematic heat acclimatization (Racinais et al., 2015) can transform these challenges into opportunities for psychological growth. This bidirectional influence exemplifies a complex adaptive system wherein psychological resources and environmental supports interact synergistically to produce emergent resilience through continuous reconstruction across multiple levels.

At the behavioral level, cognitive appraisal of thermal stress directly informs strategic choices, while the physiological and performance consequences of those choices reciprocally shape subsequent environmental perception. For example, endurance athletes employing attentional control techniques (Eysenck et al., 2007) actively filter negative somatic signals during competition. The resulting pacing strategies physically moderate core temperature accumulation (Périard et al., 2021), thereby reducing perceived environmental threat. This adaptive cycle is further supported by external resources including team coordination (Rees and Freeman, 2007) and technological aids (Cheung and McLellan, 1998), which collectively establish a socio-technical buffer that catalyzes a cascade of “psychological resource mobilization → behavioral implementation → environmental modulation.” Neurophysiological evidence confirms this interaction’s biological substrate, demonstrating that such adaptive processes strengthen prefrontal regulation of amygdala reactivity (Maren, 2001), thereby preserving executive function under stress.

In synthesis, this section advances a systems model of cognitive resilience in high-temperature environments, conceptualizing psychological adaptation as a nonlinear, dynamically coupled process across multiple levels. The proposed “Challenge Appraisal-Resource Gain” framework effectively integrates the stress-coping perspective (Lazarus and Folkman, 1984) with the “ordinary magic” resilience model (Masten, 2001). While the former establishes challenge appraisal as the initiation point of cognitive adaptation, the latter elucidates how individual resources and team support dynamically interact to sustain resilience. Together, this integrated theoretical approach systematically explains performance heterogeneity among athletes facing comparable thermal demands.

## Research hypotheses

Based on the established theoretical framework and empirical evidence, this study proposes the following research hypotheses:

Cognitive appraisal serves as the central mechanism in the stress-coping process, determining both the direction and intensity of stress responses. When athletes perceive high-temperature environments as manageable challenges rather than uncontrollable threats, they demonstrate enhanced capacity to mobilize cognitive resources and maintain functional stability (Lazarus and Folkman, 1984). Accordingly, we hypothesize:

*H1*: Challenge appraisals of high-temperature environments demonstrate stronger positive associations with cognitive resilience than do threat appraisals.

Drawing on the “Ordinary Magic” theory, which emphasizes dynamic person-environment resource interactions (Masten, 2001), the facilitative role of team support in cognitive resilience operates through a mechanism of resource-specific alignment. Empirical evidence suggests that team support differentially regulates the mobilization of psychological resources via distinct pathways. Specifically, emotional support enhances athletes’ self-efficacy and emotional security, thereby increasing both the willingness and effectiveness with which they engage and sustain emotion-regulation strategies (Freeman and Rees, 2009; Adie et al., 2012). In contrast, instrumental support reduces environmental uncertainty and decision-making demands in high-temperature contexts, establishing structured external conditions that enable flexible attentional adaptation and, consequently, optimize the allocation of attentional resources (Schinke et al., 2018). Therefore, we propose:

*H2*: Team support moderates the relationship between individual psychological resources and cognitive resilience.

*H2a*: Team support strengthens the positive association between attentional monitoring and cognitive resilience.

*H2b*: Team support enhances the positive relationship between emotional regulation and cognitive resilience.

From a developmental perspective, cognitive resilience evolves through progressive shaping of internal beliefs via accumulated experience. Longitudinal research demonstrates that with repeated heat exposure, athletes transition from compensatory regulation to proactive strategies, accompanied by strengthened self-efficacy and internalized team norms (Périard et al., 2021). Thus, we hypothesize:

*H3*: High-temperature exposure experience, self-efficacy, internalization of team norms, and cognitive resilience levels form a sequentially linked pattern consistent with theoretical expectations.

## Research design

### Participant selection and sampling strategy

This study utilized a multi-stage stratified cluster sampling method. Initially, the sampling frame was stratified into four geographical regions—North China, East China, South China, and Southwest China—based on climatic profiles and the distribution of training systems. From each stratum, two provincial-level administrative units were randomly selected, yielding a final sample of eight provinces, such as Henan, Shandong, Jiangsu, and Guangdong. Within each selected province, all qualifying athletes from provincial or national professional teams were enrolled as participants. Eligibility criteria included being aged 18 or older, possessing a minimum of 3 years of professional training, and having completed at least 200 h of high-temperature training in the preceding year. The objective of this design is to systematically examine the psychological adaptation mechanisms underlying athletes’ cognitive resilience in high-temperature environments.

To adequately test the proposed “individual-situation-organization” psychological adaptation model and ensure robust statistical power, an *a priori* sample size calculation was conducted using G*Power 3.1. With statistical power (1−*β*) set at 0.95, *α* level at 0.05, and anticipating a small-to-medium effect size based on previous research (Schmit et al., 2017), the analysis indicated a minimum requirement of 240 participants. This sample size satisfies the prerequisites for subsequent advanced statistical analyses, including structural equation modeling.

The final sample comprised 240 athletes (127 males, 52.9%; 113 females, 47.1%) aged 18–32 years (*M* = 24.3, SD = 3.6). Participants represented 12 sports disciplines with elevated heat exposure risks, categorized as endurance sports (*n* = 99, 41.3%), team sports (*n* = 86, 35.8%), and skill-based sports (*n* = 55, 22.9%). The distribution of athletic rankings included Class I (*n* = 146, 60.8%), Class II (*n* = 75, 31.3%), International Class (*n* = 5, 2.1%), and National Class (*n* = 14, 5.8%) athletes. All participants had an average of 8.6 years (SD = 2.9) of professional training experience, with cumulative heat exposure exceeding 200 h during the preceding year.

This sampling strategy—combining statistical power analysis with multiregional and multidisciplinary stratification—ensured both methodological rigor and population representativeness, thereby establishing a solid empirical foundation for the study’s conclusions.

### Measurement instruments and psychometric properties

This study established a multidimensional measurement system grounded in its theoretical framework to systematically evaluate athletes’ cognitive resilience and psychological adaptation in high-temperature environments. All instruments underwent rigorous cross-cultural adaptation and sport-specific contextualization. Structural validity was confirmed using the full sample (*N* = 240), with all fit indices meeting established psychometric standards.

Cognitive resilience was assessed using the newly developed High-Temperature Cognitive Resilience Scale, which directly measures the stability of cognitive functions maintained under thermal stress—the core adaptive outcome defined by this study’s theoretical framework. Scale development followed a systematic procedure: initial item generation (22 items) based on semi-structured interviews with 28 experienced athletes and a comprehensive review of Gucciardi et al.'s (2017) theoretical framework; Following this, five sport psychology experts were invited to appraise the content validity and contextual relevance of the preliminary items. Informed by their evaluations, six items that demonstrated substantive overlap, ambiguous phrasing, or poor situational congruence were eliminated. Subsequently, a pretest employing cognitive interviews was administered to an independent sample (*N* = 15) to evaluate item comprehensibility and semantic precision, resulting in the removal of an additional four items susceptible to interpretative ambiguity or response bias. Upon completion of this two-stage refinement procedure, a final 12-item scale was established. Its core dimensions encompass goal persistence (e.g., the volition to sustain task engagement under fatigue), attentional control (e.g., the capacity to disengage from thermal discomfort distractions), and emotion regulation (e.g., the ability to preserve affective equilibrium). Collectively, these dimensions delineate a stable pattern of cognitive adaptation exhibited by athletes in high-temperature settings.

To assess the structural validity of the outcome-defined scale, the total sample (*N* = 240) was randomly partitioned into two subsamples for cross-validation. Exploratory factor analysis (EFA) was conducted on the first subsample (*n* = 120) using principal axis factoring with Promax oblique rotation. The analysis yielded a clear three-factor solution, cumulatively explaining 67.3% of the variance, with all items exhibiting factor loadings ranging from 0.58 to 0.82 on their respective factors. Confirmatory factor analysis (CFA) was subsequently applied to the second subsample (*n* = 120). The CFA results demonstrated an acceptable model fit (*χ*^2^/df = 2.41, CFI = 0.91, RMSEA = 0.08), supporting the conceptualization of cognitive resilience as a higher-order construct comprising three correlated dimensions that collectively reflect cognitive stability. The scale exhibited strong internal consistency in the study sample, with a Cronbach’s alpha coefficient of 0.89. Composite reliability (CR) estimates for each dimension ranged from 0.84 to 0.88, while average variance extracted (AVE) values fell between 0.58 and 0.65, indicating satisfactory convergent validity. Discriminant validity was established via the heterotrait-monotrait (HTMT) ratio test, with all HTMT coefficients remaining below the conservative threshold of 0.85. Furthermore, to evaluate the scale’s temporal stability, a two-week retest was administered to a subset of 30 participants. The intraclass correlation coefficient (ICC) for the total scale score was 0.81, and ICCs for the subscales ranged from 0.76 to 0.84, demonstrating acceptable test–retest reliability. In summary, the scale demonstrates robust psychometric characteristics, thereby furnishing a reliable measurement instrument for subsequent analytical modeling.

Cognitive appraisal was measured using the challenge and threat subscales from Peacock and Wong's (1990) Stress Appraisal Scale (8 items), contextually adapted to sports environments (e.g., “high-temperature training environment” replacing “this situation”). Sample items included “I view the high-temperature training environment as a challenge” (challenge) and “I feel the high-temperature training environment is a threat to me” (threat). The 5-point Likert scale (1 = “Strongly disagree” to 5 = “Strongly agree”) demonstrated good internal consistency (*α* = 0.83).

Team support was assessed using eight core items from Freeman and Rees' (2009) social support scale, covering affective support (e.g., “When feeling unwell during high-temperature training, I receive understanding from my team”) and instrumental support (e.g., “Teammates provide practical help when I need it”). The 6-point frequency scale (1 = “Never” to 6 = “Always”) showed strong reliability (*α* = 0.86).

Supplementary measures included adapted versions of Gagge et al.'s (1967) Heat Sensation Scale (5 items) and the positive well-being subscale from Rejeski et al.’s (1991) Subjective Exercise Experience Scale (5 items). All instruments underwent standard translation-back-translation procedures, with content validity confirmed by three independent experts. Composite reliability (CR > 0.82) and average variance extracted (AVE > 0.50) met psychometric standards. Although scales employed different response formats (5- to 7-point scales), latent variable modeling ensured metric comparability in subsequent analyses. Collectively, these measures provide a psychometrically sound foundation for empirical investigation.

This research protocol received formal approval from the Ethics Review Committee of Wuxi Taihu University (Approval No. WXTC-IRB-2024-05). The study design, along with all data collection and processing procedures, adhered to the principles outlined in the Declaration of Helsinki and other pertinent ethical standards. Prior to completing the formal questionnaire, all participants were fully briefed regarding the study objectives, procedures, data utilization protocols, and confidentiality safeguards, and each provided documented informed consent. No aspect of the research involved practices detrimental to participants’ physical or psychological welfare. All data were subjected to anonymized processing and analysis throughout the study.

### Data analytic framework

This study employs a cross-sectional survey design to conduct the inaugural empirical examination of the proposed theoretical model: “Cognitive Appraisal–Resource Coupling–System Reinforcement.” While acknowledging the inherent limitations of cross-sectional data in establishing causal relationships, the statistical analyses aim to systematically investigate theoretical associations among variables, with interpretation focusing on identifying potential psychological mechanisms rather than definitive causal pathways.

A research paradigm integrating questionnaire surveys with structural equation modeling (SEM) was implemented. To balance internal and ecological validity, data collection occurred within athletes’ routine high-temperature training environments, ensuring high contextual consistency with authentic high-stress conditions—a crucial consideration for investigating real-world psychological adaptation mechanisms.

Data analysis followed a sequential approach, progressing from measurement model validation to structural model evaluation. SEM served as the core analytical framework for assessing overall model fit and examining systematic variable relationships. Complementary techniques included confirmatory factor analysis (CFA), path analysis, and bootstrap sampling. All analyses were performed using SPSS 27.0 and Mplus 8.3.

Prior to structural model construction, CFA evaluated the measurement model comprising cognitive resilience, challenge–threat appraisal, and team support. To ensure model parsimony with moderate sample sizes, dimensional scores (subscale means) served as observed indicators for latent variables. Convergent validity was assessed via composite reliability (CR > 0.70) and average variance extracted (AVE > 0.50), while discriminant validity employed the heterotrait–monotrait ratio (HTMT < 0.85). Methodological controls for common method bias included procedural remedies (e.g., anonymity assurance, reverse-scored items) and statistical verification. Harman’s single-factor test indicated the first unrotated factor accounted for 31.2% of variance, below the 40% threshold. Further validation through latent method factor analysis revealed no significant model improvement (ΔCFI = 0.012, ΔRMSEA = −0.003), with substantive path coefficients remaining unchanged, collectively indicating acceptable common method variance.

Following measurement validation, structural equation modeling tested research hypotheses. Team support’s moderating effect was examined through interaction terms (psychological resources × team support), with predictor variables centered to mitigate multicollinearity. Mediation effects involving cognitive appraisal were tested using bootstrap sampling (5,000 repetitions), with effects considered significant if 95% bias-corrected confidence intervals excluded zero—a method demonstrating robust statistical power with moderate samples.

To enhance statistical robustness, particularly for latent profile analysis (LPA), modeling utilized dimensional scores rather than individual items, effectively controlling complexity. Model selection incorporated multiple information criteria (AIC, BIC, aBIC) alongside entropy and bootstrap likelihood ratio tests (BLRT) to ensure optimal statistical and theoretical alignment. For key path coefficients, bias-corrected confidence intervals were derived from 5,000 bootstrap samples, providing distributionally robust estimates. All analyses employed two-tailed tests with *α* = 0.05.

Notably, while the *a priori* determined sample size (*N* = 240) adequately powered main and mediation effects, statistical power for exploratory complex analyses (e.g., LPA, multi-group comparisons) approached threshold levels. Consequently, analytical strategies prioritized methodological rigor: LPA determinations integrated comprehensive fit indices rather than single metrics; moderation effects emphasized effect size magnitude and theoretical meaningfulness alongside statistical significance. All findings should be interpreted as preliminary patterns requiring validation through larger-scale replication.

Regarding Hypothesis 3, which posits a sequential association from heat exposure experience through self-efficacy and team norm internalization to cognitive resilience, methodological considerations warrant emphasis. This hypothesized temporal sequence exceeds the inferential capacity of cross-sectional data. Consequently, H3 analyses assess the statistical plausibility of this theoretical progression within the measurement model, with results constituting preliminary cross-sectional evidence consistent with—but not confirming—the developmental pattern.

## Research findings

### Fundamental characteristics and correlational structure of cognitive resilience under thermal stress

To systematically investigate the distribution patterns and interrelationships of athletes’ cognitive resilience and associated psychological variables under high-temperature conditions, this study initially performed descriptive statistics and correlational analyses on core constructs. As summarized in Table 1, all variables were standardized prior to analysis. The data demonstrated acceptable normality, with absolute skewness values below 2 and kurtosis values below 7, satisfying the distributional assumptions for subsequent multivariate analyses.

**Table 1:** Descriptive statistics and correlation matrix between variables (*N* = 240).

Variables	M	SD	1	2	3	4	5	6
1. Cognitive toughness	5.35	0.71	–					
2. Challenging evaluation	5.30	0.76	0.49^**^	–				
3. Threatening evaluation	3.42	0.82	−0.35^**^	−0.30^**^	–			
4. Team support	4.58	0.49	0.22^**^	0.27^**^	−0.26^**^	–		
5. Attention flexibility	5.08	0.68	0.44^**^	0.33^**^	−0.35^**^	0.31^**^	–	
6. Emotion regulation	4.92	0.67	0.35^**^	0.19^**^	−0.17^**^	0.15^*^	0.29^**^	–

M and SD denote mean and standard deviation, respectively; ^*^p < 0.05, ^**^p < 0.01.

Within the domain of individual psychological resources, both attentional flexibility (*M* = 5.08, SD = 0.68) and emotion regulation (*M* = 4.92, SD = 0.67) exhibited moderately high scores, indicating athletes’ substantial psychological capacity in fundamental cognitive domains. The cognitive appraisal dimension revealed significant differentiation: challenge appraisal scores (*M* = 5.30, SD = 0.76) substantially exceeded threat appraisal scores (*M* = 3.42, SD = 0.82), *t*(239) = 28.74, *p* < 0.001, suggesting athletes predominantly employ positive cognitive frameworks when confronting thermal stress. Team support levels (*M* = 4.58, SD = 0.49) reflected reasonably optimal perceived social support, establishing an environmental basis for cognitive resilience development.

The correlation matrix revealed theoretically meaningful covariation patterns. Cognitive resilience demonstrated a significant positive correlation with challenge appraisal (*r* = 0.49, *p* < 0.01) and a negative association with threat appraisal (*r* = −0.35, *p* < 0.01), consistent with cognitive appraisal theory’s proposition regarding differential relationships between appraisal orientations and adaptation outcomes. Team support correlated positively with cognitive resilience (*r* = 0.22, *p* < 0.01), showing a stronger association with challenge appraisal (*r* = 0.27, *p* < 0.01) than with threat appraisal (*r* = −0.26, *p* < 0.01), indicating social support systems may indirectly foster resilience through promoting challenge-oriented appraisals.

Among individual psychological resources, both attentional flexibility (*r* = 0.44, *p* < 0.01) and emotion regulation (*r* = 0.35, *p* < 0.01) maintained moderate positive correlations with cognitive resilience. The significant correlation between these resources (*r* = 0.29, *p* < 0.01) suggests potential synergistic effects during thermal adaptation. Cross-sport comparisons revealed endurance athletes surpassed team-sport athletes in both cognitive resilience (*M* = 5.42, SD = 0.68 vs. *M* = 5.18, SD = 0.74) and challenge appraisal (*M* = 5.45, SD = 0.79 vs. *M* = 4.98, SD = 0.66), with independent *t*-tests confirming statistically significant group differences (*t* = 2.58, *p* = 0.011; *t* = 3.45, *p* = 0.001, respectively).

All correlation coefficients remained within acceptable ranges, and variance inflation factors below 2.0 precluded multicollinearity concerns. These foundational results provide crucial empirical grounding for investigating psychological adaptation mechanisms of cognitive resilience in high-temperature environments, with the observed correlation patterns aligning with theoretical expectations and establishing a methodological basis for subsequent complex variable relationship analyses.

### Psychological mechanisms of cognitive resilience: the mediating role of challenge appraisals

To examine the mediating role of challenge appraisal in the relationship between psychological resources and cognitive resilience, this study employed structural equation modeling (SEM). Initial confirmatory factor analysis established adequate psychometric properties for all latent variables: composite reliability (CR = 0.82–0.91) and average variance extracted (AVE = 0.54–0.68) exceeded recommended thresholds, while heterotrait-monotrait ratios (HTMT < 0.85) confirmed discriminant validity, establishing a solid foundation for subsequent structural analysis.

The theoretical model demonstrated excellent data fit: *χ*^2^/df = 2.36, CFI = 0.92, TLI = 0.90, RMSEA = 0.075 (90% CI [0.065, 0.085]), and SRMR = 0.045, collectively indicating strong alignment between the hypothesized model and observed data.

Path coefficients revealed that challenge appraisal significantly predicted cognitive resilience (*β* = 0.38, *p* < 0.001), supporting H1. Both attentional flexibility (*β* = 0.28, *p* < 0.001) and emotion regulation (*β* = 0.22, *p* = 0.002) demonstrated direct effects on resilience. Furthermore, these psychological resources significantly predicted challenge appraisal (attentional flexibility: *β* = 0.39, *p* < 0.001; emotion regulation: *β* = 0.26, *p* < 0.001) (Table 2).

**Table 2:** Standardized path coefficients and mediation effects analysis.

Paths	*β*	SE	*p*-value	95% Boot CI
Direct path				
Attentional flexibility → cognitive resilience	0.28	0.06	<0.001	[0.16, 0.40]
Emotion regulation → cognitive resilience	0.22	0.07	0.002	[0.08, 0.36]
Challenging evaluation → cognitive resilience	0.38	0.05	<0.001	[0.28, 0.48]
Indirect route				
Attentional flexibility → challenging evaluations → cognitive resilience	0.15	0.03	–	[0.09, 0.22]
Emotion regulation → challenging appraisal → cognitive resilience	0.10	0.03	–	[0.05, 0.16]

*N* = 240. Bootstrap 5,000 sampling. All confidence intervals are bias-corrected.

Bootstrap analysis (5,000 samples) confirmed significant indirect effects: attentional flexibility influenced cognitive resilience through challenge appraisal (effect = 0.15, 95% CI [0.09, 0.22]), as did emotion regulation (effect = 0.10, 95% CI [0.05, 0.16]). The model explained substantial variance in both challenge appraisal (*R*^2^ = 0.42) and cognitive resilience (*R*^2^ = 0.51). Proportionally, challenge appraisal mediated 34.8% of attentional flexibility’s total effect and 31.3% of emotion regulation’s total effect on cognitive resilience.

These findings support challenge appraisal as a significant mediator between psychological resources and cognitive resilience. Theoretically, attentional flexibility facilitates reinterpretation of physiological arousal as adaptive preparation rather than performance threat, while emotion regulation maintains task-directed cognitive resources by stabilizing affective responses to thermal discomfort.

This study elucidates the pivotal role of cognitive appraisal in connecting psychological resources to environmental adaptation. As a core mechanism, challenge appraisal demonstrates how individuals transform physiological stress into manageable demands through cognitive restructuring. These findings not only extend cognitive appraisal theory to extreme environments but also suggest that heat adaptation training should incorporate cognitive interventions to develop psychological patterns that convert environmental pressures into performance opportunities, ultimately enabling the transition from passive endurance to active adaptation.

### Heterogeneity in cognitive resilience: a latent profile analysis

This study employed latent profile analysis (LPA) to investigate the heterogeneous structure of athletes’ cognitive resilience in high-temperature environments. Using standardized scores from three core dimensions—goal persistence, attentional control, and emotional regulation—we systematically compared models specifying 2–5 latent profiles. Model selection was based on comprehensive fit indices (Table 3), including the Akaike information criterion (AIC), Bayesian information criterion (BIC), adjusted BIC (aBIC), entropy, and the bootstrap likelihood ratio test (BLRT).

**Table 3:** Comparison of fitted metrics for potential profile analysis models of cognitive toughness (*N* = 240).

Modelling	AIC	BIC	aBIC	Entropy	BLRT *p* value	Category probability
Category 2	1,325.48	1,362.15	1,298.73	0.82	<0.001	0.63/0.37
Category 3	1,248.92	1,295.89	1,220.15	0.84	<0.001	0.42/0.35/0.23
Category 4	1,212.36	1,258.74	1,198.53	0.86	<0.001	0.38/0.29/0.21/0.12
Category 5	1,198.25	1,254.93	1,172.39	0.85	0.124	0.35/0.27/0.18/0.12/0.08

Smaller values of AIC, BIC, and aBIC indicate better model fit; entropy values closer to 1 indicate higher classification accuracy; BLRT is significant indicating that the k-category model outperforms the k-1 category model. The optimal model is shown in bold.

The four-profile solution demonstrated optimal statistical properties across multiple indices. This model exhibited the lowest AIC, BIC, and aBIC values, with high classification accuracy (entropy = 0.86). BLRT results further confirmed its superiority, as the five-profile model failed to reach statistical significance and produced a marginal profile with probability below 10%. Consequently, the four-profile model was selected as the optimal representation of cognitive resilience heterogeneity.

**Figure 2:**
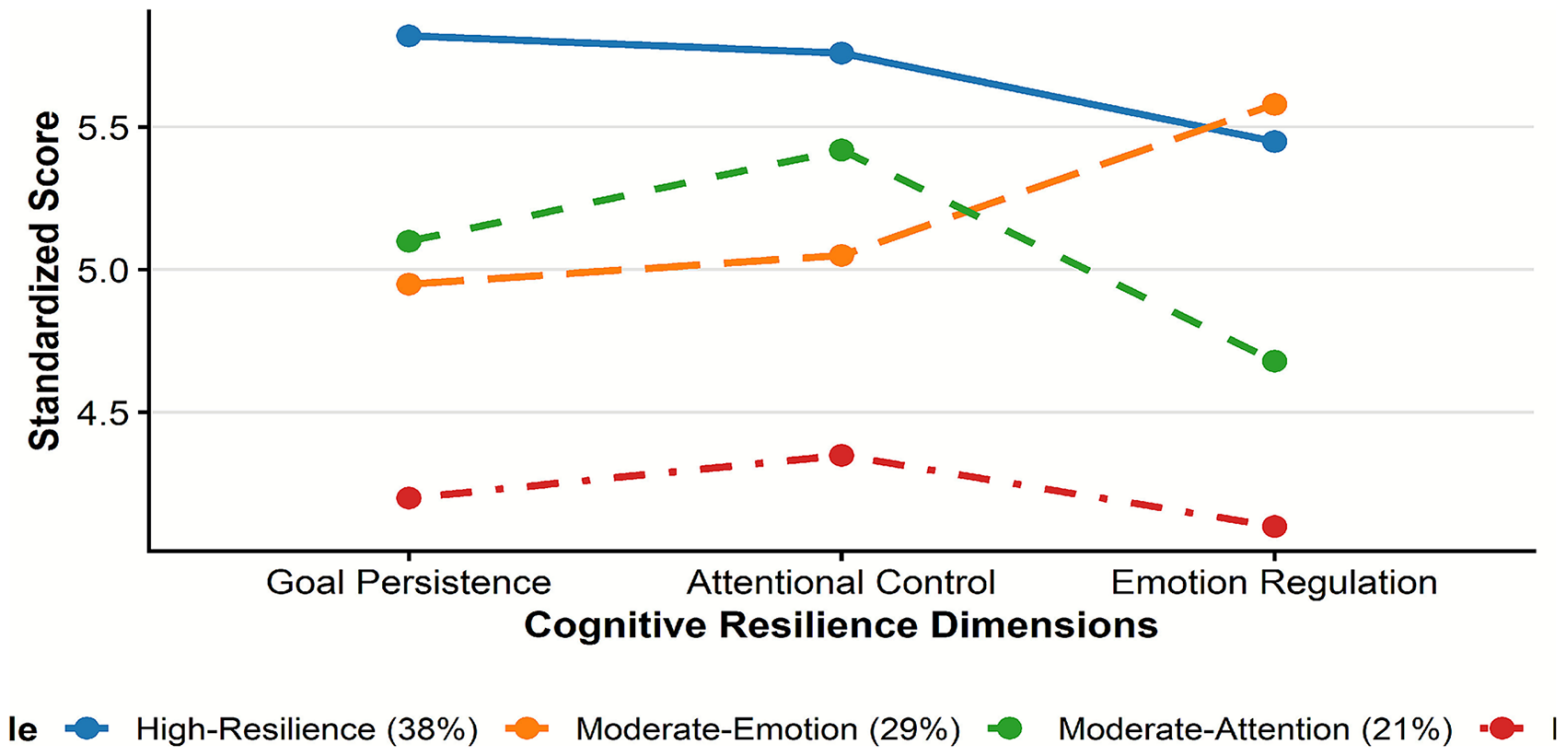
Characterisation of the four-category model for potential profiling of cognitive resilience. The figure illustrates the mean profile characteristics of the four potential categories on the three standardized dimensions of cognitive resilience.

The four latent profiles exhibited distinct dimensional configurations (Figure 2). Profile 1 (38%; High-Resilience Type) displayed superior performance across all dimensions: goal persistence (*M* = 5.82, SD = 0.41), attentional control (*M* = 5.76, SD = 0.38), and emotional regulation (*M* = 5.45, SD = 0.43). Profile 2 (29%; Emotion-Goal Vulnerable Type) demonstrated relatively preserved emotional regulation (*M* = 5.58, SD = 0.45) but compromised goal persistence (*M* = 4.95, SD = 0.52). Profile 3 (21%; Attention-Emotion Vulnerable Type) showed adequate attentional control (*M* = 5.42, SD = 0.49) but the lowest emotional regulation capacity (*M* = 4.68, SD = 0.55). Profile 4 (12%; Low-Resilience Type) scored significantly lower across all three dimensions.

Latent profile analysis yielded four discrete profiles, among which the low-resilience category constituted the smallest subgroup, representing 12% of the sample (*n* ≈ 29). To assess the reliability of subsequent subgroup comparisons, a post-hoc statistical power analysis was performed. Using G*Power 3.1 with *α* = 0.05 and a moderate effect size (*f* = 0.25), the post-hoc power for a one-way ANOVA involving this minimal subgroup was approximately 0.58, falling below the conventional benchmark of 0.80. This result suggests a limited capacity to detect between-group differences within this category.

To improve analytical robustness without compromising theoretical coherence, the two intermediate resilience profiles—emotion–goal vulnerability and attention–emotion vulnerability—were consolidated into a unified category termed “moderate resilience with structural imbalance,” which collectively accounted for 50% of the sample. A follow-up multi-group moderation analysis under this reconfigured schema demonstrated stable model fit indices (ΔCFI < 0.01), and the moderating effect of team support retained statistical significance (*β* = 0.18, *p* < 0.05), confirming that the merger did not substantively alter the principal findings.

Notwithstanding, the moderation effects observed within smaller subgroups—notably the low-resilience profile—should be interpreted with caution, as the stability of these parameter estimates necessitates verification in larger-scale samples.

External validation analyses revealed meaningful group differences. The high-resilience profile contained disproportionately more international- and national-level athletes (*χ*^2^ = 35.72, *p* < 0.001) and reported significantly greater annual heat exposure training, *F* (3, 236) = 15.88, *p* < 0.001. Cognitively, this group exhibited the most pronounced challenge appraisal orientation and minimal threat appraisal. The emotion-goal vulnerable profile was overrepresented among team-sport athletes, while the attention-emotion vulnerable profile correlated with fewer years of training experience.

These findings demonstrate that cognitive resilience manifests through qualitatively distinct multidimensional patterns rather than existing along a unitary continuum. The identification of these heterogeneous profiles provides an empirical foundation for developing differentiated psychological interventions. Crucially, this heterogeneity raises important questions regarding the generalizability of psychological mechanisms: does the team support resource gain effect operate consistently across different cognitive resilience profiles? Subsequent analyses will address this question by examining profile membership as a potential moderator of team support mechanisms.

### Contextual enhancement mechanisms: testing the moderating role of team support

This study employed hierarchical regression analysis to systematically examine the moderating role of team support in the relationship between psychological resources and cognitive resilience. Consistent with our theoretical framework, we hypothesized that team support would enhance the positive association between individual psychological resources and cognitive resilience. All predictor variables were mean-centered prior to analysis, and variance inflation factors remained below 2.0, confirming the absence of multicollinearity concerns.

The hierarchical regression results (Table 4) demonstrated that after controlling for the main effects of attentional flexibility and emotion regulation (collectively explaining 38% of variance), the addition of team support significantly improved model fit (Δ*R*^2^ = 0.04, *p* < 0.01). Most importantly, including the interaction terms further enhanced model explanatory power to 47% (Δ*R*^2^ = 0.05, *p* < 0.001). Both the attentional flexibility × team support interaction (*β* = 0.18, *p* < 0.01) and emotion regulation × team support interaction (*β* = 0.15, *p* < 0.05) reached statistical significance, supporting the hypothesized moderating role of team support.

**Table 4:** Analysis results of the moderating effect of team support.

Predictor variables	Model 1	Model 1	Model 1
Step 1: main effects			
Attention flexibility	0.44^***^	0.43^***^	0.42^***^
Emotional regulation	0.35^***^	0.34^***^	0.33^***^
Team support		0.21^**^	0.20^**^
Step 2: interaction effects			
Attention flexibility × team support			0.18^**^
Emotional regulation × team support			0.15^*^
*R* ^2^	0.38	0.42	0.47
Δ*R*^2^		0.04^**^	0.05^***^

*N* = 240; ^*^p < 0.05, ^**^p < 0.01, ^***^p < 0.001.

Simple slope analyses elucidated the specific nature of these interaction effects. Under high team support conditions, the relationship between attentional flexibility and cognitive resilience was significantly stronger (*β* = 0.52, *p* < 0.001) compared to low support conditions (*β* = 0.32, *p* < 0.01), with this difference reaching statistical significance (*t* = 2.89, *p* < 0.01). A similar pattern emerged for emotion regulation, demonstrating a stronger association with cognitive resilience in high-support environments (*β* = 0.41, *p* < 0.001) versus low-support conditions (*β* = 0.25, *p* < 0.01; *t* = 2.34, *p* < 0.05).

Building upon the latent profile analysis findings, we further examined moderation effect heterogeneity across cognitive resilience subtypes. Multi-group analysis revealed significant between-profile differences in the team support enhancement effect (Δ*χ*^2^ = 15.36, *p* < 0.01). Specifically, the moderating effect was most pronounced in the low-resilience profile (*β*_interaction = 0.31, *p* < 0.001), moderately evident in both medium-resilience profiles (emotion-goal vulnerable: *β*_interaction = 0.17, *p* < 0.05; attention-emotion vulnerable: *β*_interaction = 0.19, *p* < 0.05), and non-significant in the high-resilience profile (*β*_interaction = 0.08, *p* = 0.21).

These findings collectively demonstrate that team support functions as a crucial contextual resource that synergistically interacts with individual psychological resources. The data confirm that team support strengthens the positive association between psychological resources and cognitive resilience, while revealing important heterogeneity in this enhancement effect across distinct resilience profiles. These results provide valuable insights for developing targeted psychological interventions tailored to athletes’ specific resilience characteristics.

## Discussion

### The mediating role of challenge appraisal: cognitive restructuring as a Core mechanism in psychological adaptation

This study confirms the complete mediation of challenge appraisal in the relationship between attentional flexibility, emotion regulation, and cognitive resilience. It demonstrates that psychological adaptation to high-temperature environments operates through a meaning-transformation process centered on cognitive reappraisal, rather than through the direct transfer of fundamental psychological resources (Lazarus and Folkman, 1984).

Specifically, individual psychological resources initially facilitate the transformation of primary appraisal under thermal stress. Attentional flexibility allows athletes to continuously monitor physiological signals—such as heart rate and core temperature—without lapsing into cognitive fixation (Shibasaki et al., 2017), thereby supplying accurate environmental data for appraisal. Concurrently, emotion regulation buffers the anxiety elicited by thermal discomfort (Gross, 2015), establishing a stable psychological foundation for subsequent cognitive reframing. The synergistic operation of these capacities supports athletes in reinterpreting high-temperature conditions from a “performance threat” into a “manageable challenge” during secondary appraisal (Tomaka et al., 1997).

Such cognitive reappraisal in turn drives a strategic reallocation of cognitive resources. When thermal stress is appraised as a challenge, attentional and executive resources otherwise devoted to threat monitoring—such as tracking discomfort or anticipating failure—are liberated and redeployed toward adaptive functions, including goal maintenance, tactical planning, and motor execution. At the level of cognitive processing, this mechanism explains how athletes preserve functional stability under comparable thermal loads. Essentially, the behavioral expression of cognitive resilience stems from an optimized redistribution of available cognitive resources.

These findings reposition challenge appraisal from a relatively static cognitive predisposition to a pivotal regulatory hub within the dynamic adaptive process. They indicate that under high-temperature stress, interindividual variation in performance is determined not by the magnitude of physiological reactivity, but by the subjective construction of meaning assigned to stress signals (Lazarus and Folkman, 1984). Consequently, psychological training in high-temperature contexts should shift its emphasis from merely bolstering basic cognitive capacities toward systematically cultivating metacognitive skills that enable the reframing of physiological arousal as task-relevant challenge cues.

In summary, the mediating function of challenge appraisal affirms the centrality of meaning construction in cognitive resource allocation and delineates the intrinsic psychological pathway through which cognitive resilience develops. This provides a critical theoretical and empirical basis for designing heat-adaptation training protocols grounded in cognitive reappraisal—for instance, through simulated scenarios and guided feedback that reinforce adaptive interpretive schemas such as “thermal stress as performance opportunity.”

### The enhancing effect of team support: interactive dynamics between individual resources and contextual factors

The findings of this study demonstrate that team support functions as a significant positive moderator in the relationship between individual psychological resources—specifically attentional flexibility and emotion regulation—and cognitive resilience. This moderating effect is especially pronounced among athletes with lower baseline resilience. These results suggest that environmental support and individual resources interact through a dynamic coupling mechanism, jointly facilitating the development of cognitive resilience (Masten, 2001).

Analysis of the moderation effect indicates that elevated levels of team support amplify the predictive capacity of individual psychological resources on cognitive resilience. In settings characterized by adequate team support, attentional flexibility is more effectively channeled into adaptive monitoring of thermal cues, as opposed to rigid threat fixation. Similarly, emotion regulation capabilities are better sustained in preserving affective equilibrium, thereby mitigating the risk of being overwhelmed by thermal distress. This facilitative role arises from two complementary mechanisms underpinning team support: first, structured operational protocols—such as synchronized hydration and rotation schedules—diminish environmental unpredictability, which in turn liberates cognitive resources from incessant monitoring demands (Rees and Freeman, 2007); second, emotional support and the internalization of collective norms bolster individuals’ efficacy beliefs and behavioral perseverance through processes of social reinforcement.

Notably, the moderating influence of team support displays considerable between-group heterogeneity. It is most robust among low-resilience athletes, whereas it fails to reach significance in high-resilience counterparts. This divergent pattern implies that team support operates primarily in a compensatory capacity rather than conferring uniform enhancement. For athletes with comparatively constrained psychological resources, team support supplies external scaffolding that offsets deficits in self-regulatory capacity. In contrast, for high-resilience athletes who already exhibit well-developed self-regulation, the marginal utility of environmental support appears attenuated. This observation resonates with Conservation of Resources Theory (Hobfoll, 1989), which proposes that external support exerts a more substantial protective effect when internal resources are scarce.

From an applied perspective, these outcomes underscore the importance of implementing differentiated support strategies. For low-resilience athletes, support should be explicit, structured, and multifaceted—encompassing clearly defined thermal coping protocols, timely empathetic feedback, and tangible instrumental aid. For high-resilience athletes, emphasis may be placed on sustaining an ambient supportive climate while safeguarding sufficient latitude for autonomous self-regulation. Such tailored, resilience-informed approaches hold promise for optimizing team environmental returns under conditions of limited resources.

In summary, team support does not act as an isolated contextual factor; rather, it co-shapes cognitive resilience via dynamic interplay with individual resources. This insight advances the current understanding of psychological adaptation mechanisms in high-temperature settings and highlights the imperative of cultivating synergistic frameworks in athletic training—ones that deliberately align environmental provisions with individual psychological profiles.

### The heterogeneous architecture of cognitive resilience: multidimensional capacity configurations and their implications

Employing latent profile analysis, this study delineates four distinct heterogeneous profiles of cognitive resilience among athletes in high-temperature environments: high resilience, emotion–goal vulnerability, attention–emotion vulnerability, and low resilience. These outcomes furnish empirical evidence for comprehending the complex manifestations of cognitive resilience under specific stress conditions, thereby engaging critically with conventional measurement paradigms that conceptualize resilience predominantly as a continuous and homogeneous trait (Gucciardi et al., 2015).

The detection of two intermediate profiles—emotion–goal vulnerability and attention–emotion vulnerability—reveals possible asynchrony in the development across dimensions of cognitive resilience. This insight enriches current understanding of the construct’s internal architecture, indicating that under acute stress it assumes multidimensional combinatorial patterns distinct from those observed in normative contexts. The emotion–goal vulnerability profile reflects a decoupling between emotion regulation capacity and goal-persistence behaviors, implying that regulatory advantages may not reliably translate into sustained goal-directed action under specific situational demands—an observation congruent with Locke and Latham’s (2002) theoretical work on goal internalization mechanisms. Conversely, the attention–emotion vulnerability profile underscores the foundational role of emotion regulation in supporting cognitive function. Its theoretical consonance with predictions derived from Eysenck et al.’s (2007) attentional control theory under stress conditions is notable, and it demonstrates particular explanatory relevance within the context of thermally induced physiological strain.

By shifting focus from global resilience levels to the functional implications of specific cognitive dimension configurations, this research extends beyond traditional analytical boundaries. The refined theoretical perspective presented here aligns strongly with the context-specific resilience framework articulated by Fletcher and Sarkar (2013), illuminating how stressor properties actively shape patterns of psychological adaptation.

Practically, these findings offer explicit guidance for designing psychological interventions in high-temperature settings. The identification of discrete cognitive resilience profiles argues for moving beyond standardized training protocols toward differentiated intervention systems tailored to individual capability structures. Intervention priorities must be systematically calibrated: for the emotion–goal vulnerability profile, enhancing goal-management competencies is paramount, whereas the attention–emotion profile warrants integrated training targeting emotion-attention synergy. Such precision intervention paradigms, anchored in empirically derived profiles, establish a theoretical rationale for optimizing the efficacy of psychological support under resource-limited conditions.

It should be acknowledged that the latent profile analysis adopted herein provides methodological leverage for examining group heterogeneity in cognitive resilience, partially mitigating the limitations inherent in conventional variable-centered approaches for detecting subgroup characteristics. Future investigations would benefit from longitudinal designs to trace the dynamic trajectories of these heterogeneous profiles and to elucidate their intrinsic linkages with behavioral outcomes through multimodal assessment. Such endeavors would propel the field toward more nuanced and ecologically grounded research pathways.

### Integrative theoretical interpretation: from critical pathways to a comprehensive framework

The present study substantiates the theoretical integrative value of the “challenge appraisal-resource gain” model, which extends beyond confirming the causal pathway from individual resources to cognitive resilience. It also transcends the long-standing dichotomy between “individual traits” and “environmental influences” (Masten, 2018). In contrast to approaches that conceptualize cognitive resilience as a stable trait (Gucciardi et al., 2015), this study demonstrates—through the mediating role of challenge appraisal and the moderating effect of team support—that cognitive resilience constitutes an emergent property shaped through dynamic psychological processes within specific environmental contexts.

A pivotal contribution of the model is its reconceptualization of challenge appraisal as a dynamic cognitive-regulatory process, rather than a static psychological variable. This shifts away from perspectives that treat appraisal primarily as an initial stage of stress response (Lazarus, 1991). The findings indicate that sustaining a challenge appraisal itself demands continuous attentional monitoring and emotional regulation (Shibasaki et al., 2017), thereby advancing the understanding of stress appraisal mechanisms and offering an explanatory lens for performance variability among athletes under comparable pressure conditions.

Moreover, the model elucidates the precise mechanisms through which environmental support modulates psychological adaptation pathways. Moving beyond macro-level discussions that position the environment merely as a contextual backdrop (Masten, 2001), this investigation demonstrates that team support moderates the functional transformation from resources through appraisal to resilience, with especially salient benefits among individuals with lower baseline resilience. This outcome diverges from perspectives attributing a generalized role to social support, revealing instead that support effects are contingent on subgroup characteristics—an insight critical for developing targeted interventions.

Heterogeneous profiles identified via latent profile analysis further validate the explanatory scope of the model. The existence of distinct cognitive resilience subtypes challenges conventional measurement paradigms that treat resilience as a unidimensional continuum (Gucciardi et al., 2015). The simultaneous presence of such heterogeneity alongside a core mediating pathway requires an explanatory framework capable of accommodating both mechanistic specificity and phenotypic diversity. By integrating core transformation mechanisms with contextual moderators, the proposed model addresses this theoretical necessity.

From an applied standpoint, this study advances an actionable, multi-tiered intervention framework. It delineates challenge appraisal as a focal intervention target while emphasizing the pivotal role of environmental support in calibrating intervention efficacy. This integrated emphasis on core mechanisms and contextual variability establishes an evidence-based rationale for deploying individualized psychological interventions in demanding environments, such as high-temperature athletic settings.

In summary, the distinctive merit of the model resides in its capacity to provide a systematic explanatory framework for heterogeneous outcomes under homogeneous environmental conditions. Rather than prioritizing either individual or environmental factors, it delineates their interaction within specific psychological processes, thereby furnishing a more nuanced theoretical instrument for examining adaptation within complex ecologies.

## Research findings and recommendations

### Research findings

Through the development and validation of the “Challenge Appraisal-Resource Gain” theoretical model, this study systematically elucidates the psychological adaptation mechanisms underlying athletes’ cognitive resilience in high-temperature environments. The principal findings are as follows:

First, challenge appraisal serves as the core cognitive transformation mechanism in resilience development. Functioning as a primary stress-coping process, this appraisal reconstructs the subjective meaning of thermal stress by reinterpreting physiological signals as manageable developmental challenges. This cognitive restructuring facilitates the reallocation of cognitive resources from emotional processing to task-oriented operations, thereby extending the explanatory scope of cognitive appraisal theory in athletic stress contexts.

Second, the systemic coupling of individual psychological resources and team support establishes the ecological foundation for resilience development. Team support not only mitigates environmental uncertainty through social buffering mechanisms but, more critically, enhances individual resource mobilization efficiency via complementary resource effects. This dynamic person-environment interaction reveals the socio-ecological nature of cognitive resilience formation.

Third, the heterogeneous structure of cognitive resilience reflects multiple psychological adaptation pathways. The identification of four distinct latent profiles demonstrates that dimensional psychological capacities can achieve functional stability through differentiated configurations. This “equifinality” phenomenon challenges conventional linear developmental perspectives and provides a theoretical basis for precision intervention models accommodating individual differences.

Fourth, the integrated model achieves methodological innovation through theoretical synthesis. By incorporating micro-level cognitive restructuring, meso-level resource enhancement, and macro-level typological differentiation within a unified framework, this model transcends single-level analytical limitations and establishes a systematic paradigm for investigating psychological mechanisms in extreme environmental performance.

### Practical recommendations

Based on the “Challenge Appraisal-Resource Gain” model, we propose four evidence-based recommendations to enhance athletes’ thermal adaptation:

First, institutionalize challenge appraisal training as the cornerstone of psychological heat adaptation. Implementation should systematically cultivate cognitive reframing habits through structured integration of reappraisal techniques into thermal training. For instance, guide athletes to reconstruct “tachycardia” as “metabolic activation” and reinterpret “thermal stress” as “competitive resilience development.” Strengthen these cognitive patterns through biofeedback and scenario simulation to establish durable psychophysiological connections and optimize cognitive resource allocation.

Second, develop multidimensional team support systems to activate resource enhancement effects. Transform team support from generalized encouragement into precise resource provision by establishing dual-assistance networks encompassing affective support (e.g., empathetic communication) and instrumental support (e.g., optimized heat-rotation protocols). Prioritize structured assistance for low-resilience athletes to maximize environmental compensation effects, effectively reducing uncertainty and liberating cognitive resources for task execution.

Third, implement precision interventions based on cognitive resilience typologies. Replace uniform psychological training with customized approaches informed by profile assessment:

For emotion-goal vulnerable athletes: enhance goal-setting and motivational maintenance.

For attention-emotion vulnerable individuals: strengthen emotional regulation to conserve attentional resources.

For low-resilience subgroups: initiate comprehensive capacity-building programs.

Fourth, establish multidisciplinary intervention frameworks guided by the integrated model. Recognize thermal adaptation as a systemic endeavor requiring cross-disciplinary integration. Systematically incorporate cognitive restructuring, resource enhancement, and precision intervention strategies into routine thermal training and competition preparation. Foster collaborative teams involving coaches, sport psychologists, physicians, and administrators to ensure coordinated implementation across physiological monitoring, tactical planning, and psychological adjustment phases, collectively constructing optimal ecosystems for cognitive resilience development.

## Research limitations and future directions

This study has the following main limitations. From a methodological perspective, although the cross-sectional design effectively identifies associations among variables, it does not permit the examination of the dynamic developmental trajectory of cognitive resilience within high-temperature contexts. Regarding sampling, the limited size of the smallest subgroup identified via latent profile analysis reduces statistical power, which may affect the robustness of moderation estimates specific to this profile. In terms of measurement, the predominant reliance on self-reported data restricts the ability to incorporate objective physiological indices and behavioral performance measures, thereby constraining a more nuanced interpretation of the psychophysiological mechanisms underlying adaptive processes. While post-hoc analytic strategies—such as profile merging and power analysis—were implemented to strengthen the stability of subgroup comparisons, findings derived from smaller subgroups should be considered preliminary, and their generalizability warrants verification in future studies.

Future investigations could extend this work in several meaningful directions: utilizing longitudinal or experience-sampling methodologies to capture the temporal dynamics of cognitive resilience; examining the generalizability of the proposed theoretical model across diverse athletic disciplines and skill levels; and adopting multimodal assessment frameworks that concurrently monitor psychological, physiological, and behavioral indicators. Such integrative approaches would facilitate the development of a coherent theoretical architecture that bridges macro-level performance outcomes with underlying micro-level processes, collectively guiding high-temperature sport psychology toward more precise and ecologically valid inquiry.

## Data Availability

The raw data supporting the conclusions of this article will be made available by the authors, without undue reservation.
